# Long noncoding RNA SNHG4 promotes the malignant progression of hepatocellular carcinoma through the miR‐211‐5p/CREB5 axis

**DOI:** 10.1002/cam4.5559

**Published:** 2022-12-23

**Authors:** Jiannan Qiu, Peng Wang, Zheng Chen, Yan Zhou, Guang Zhang, Zhongxia Wang, Junhua Wu, Qiang Zhu, Chunping Jiang

**Affiliations:** ^1^ Department of Hepatobiliary Surgery Affiliated Drum Tower Hospital, Medical School of Nanjing University Nanjing China; ^2^ Children's Hospital of Nanjing Medical University Nanjing China; ^3^ Jinan Microecological Biomedicine Shandong Laboratory Jinan City Shandong Province China; ^4^ State Key Laboratory of Pharmaceutical Biotechnology, National Institute of Healthcare Data Science at Nanjing University, Jiangsu Key Laboratory of Molecular Medicine, Medical School of Nanjing University, Nanjing University Nanjing Jiangsu China

**Keywords:** CREB5, hepatocellular carcinoma, malignancy, miR‐211‐5p, SNHG4

## Abstract

**Background and Aims:**

Hepatocellular carcinoma (HCC) is one of the main death‐leading malignant tumors which deserve in‐depth explorations to uncover the underlying molecular mechanisms. Plenty of proofs have revealed that long noncoding RNAs (lncRNAs) participate in malignancy and progression of HCC. Nevertheless, the definite role of lncRNA‐SNHG4 in HCC remains vague.

**Methods:**

To figure out the role of SNHG4 in HCC, the bioinformatics analysis and functional assays and in vivo assay were performed.

**Results:**

Our findings demonstrated that the data from The Cancer Genome Atlas (TCGA) displayed that the higher expression of lncRNA SNHG4 was detected in HCC tissues, which predicted the poor prognosis. The upregulation of SNHG4 was positively associated with worse clinicopathological characteristics. The functional experiments were performed to identify the role of SNHG4 in HCC. We found that SNHG4 enhanced the proliferative, migratory and invasive capacities of HCC cell line, and facilitated the tumor growth in vivo. A series of follow‐up studies have shown that SNHG4 promoted the progression and malignancy of HCC through upregulating CREB5 via sponging miR‐211‐5p.

**Conclusion:**

Collectively, the above findings suggest that SNHG4 promotes HCC malignancy through the SNHG4/miR‐211‐5p/CREB5 axis, providing potential therapeutic targets and prognostic factors for HCC.

**Highlights**

- SNHG4 is overexpressed in HCC and correlated with the poor clinical characteristics
- SNHG4 promotes the malignant progression of HCC by reducing miR‐211‐5p expression
- MiR‐211‐5p inhibits CREB5 expression in HCC
- The oncogenic effect of SNHG4 in HCC can be reversed by CREB5 silencing

## Graphical abstract

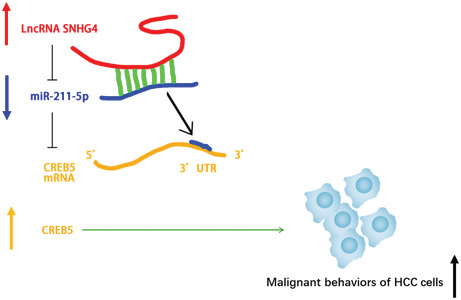

## 1 INTRODUCTION

Hepatocellular carcinoma (HCC), as one of the most prevalent malignancies, has attracted the attention of researchers all over the world. Although, with the multifaceted development of modern surgical techniques, a growing number of therapeutic methods have been applied to the treatment of HCC, partial hepatectomy and liver transplantation remain the crucial therapies for HCC.^1^, ^2^ Given that most patients are diagnosed with advanced‐stage HCC, there is often a lack of opportunity for surgery, which results in limited therapeutic outcomes for HCC patients. Meanwhile, the high morbidity and mortality of HCC in China has become a heavy burden on the society. Accordingly, exploring the mechanism of the occurrence and development of HCC is an urgent task for all liver disease researchers.

Long noncoding RNAs (lncRNAs), as noncoding transcripts with a length of over 200 nucleotides, could interact with multiple molecules to play a crucial role in regulating gene expression and affecting cellular function.^3^, ^4^ In recent studies, many findings have clarified the complex and diverse role of lncRNAs in the tumorigenesis and progression of HCC.^5^, ^6^ In view of the multiple effect of lncRNAs in tumors, it was widely reported that lncRNAs was involved with cellular proliferation, metastasis, ferroptosis, stemness, chemoresistance and so on.^7^, ^8^, ^9^, ^10^ For instance, the upregulation of lncRNA NEAT1 plays a novel and essential role in promoting ferroptosis via regulating miR‐362‐3p and MIOX in HCC.^11^ Long noncoding RNA PAARH accelerated the development in HCC through upregulating HOTTIP and enhanced angiogenesis through interacting with HIF‐1α which subsequently promoted VEGF expression.^12^ LncRNA DUBR enhanced stem cell phenotype and oxaliplatin‐resistance in HCC via upregulating CIP2A expression through E2F1‐mediated transcriptional regulation and competingly binding with miR‐520d‐5p.^13^

Small nucleolar host gene 4 (SNHG4), identified as a lncRNA, belongs to the SNHGs family. It has been reported that the SNHG4 is involved in regulating the occurrence and progression of multiple tumors.^14^, ^15^, ^16^ Numerous studies have revealed that lncRNA SNHG4 was involved with the progression of colorectal cancer,^17^ non‐small cell lung cancer,^18^ osteosarcoma,^19^ renal cell carcinoma^20^ and so on. In brief, SNHG4 enhanced the malignancy and immune escape of colorectal cancer via upregulating MET through inhibiting miR‐144‐3p.^17^ SNHG4 served as one prognostic factor and accelerated the progression of osteosarcoma via sponging miR‐224‐3p.^19^ Whereas, the precise mechanism of lncRNA SNHG4 in the progression of HCC remains unclear.

Our findings proved that lncRNA SNHG4 expression was upregulated in HCC tissues and cell lines. HCC patients with high expression of SNHG4 are characterized by a poor prognosis. Meanwhile, we observed that higher SNHG4 expression was remarkably related with larger tumor size, microvascular invasion, and higher TNM and Edmonson stages. The functional experiments revealed that SNHG4 overexpression resulted in the enhanced cellular malignant behaviors. After plenty of assays for exploring the underlying mechanism, we discovered that SNHG4 promoted the progression and malignancy of HCC through upregulating CREB5 expression via sponging miR‐211‐5p. Altogether, our study uncovered that SNHG4/miR‐211‐5p/CREB5 axis facilitated the malignancy and progression of HCC.

## 2 METHODS AND MATERIALS

### 2.1 Cell culture and patient samples

The HepG2, Huh7, Hep3B, LM3, MHCC‐97H, and L02 cell lines were obtained from the American Type Culture Collection (Manassas, VA, USA). Cells were cultured with DMEM (Gibco, USA) which comprising 10% fetal bovine serum (FBS) and 1% antibiotics (streptomycin/penicillin; Gibco, USA) at 37°C, 5% CO_2_ in a humidified incubator.

Liver samples from 60 HCC patients who underwent hepatic partial resection at the Hepatobiliary Center of the Affiliated Drum Tower Hospital of Medical School of Nanjing University were partly frozen in liquid nitrogen and the rest were stored in formaldehyde solution for subsequent research. The consent of the Ethics Committee of the Affiliated Drum Tower Hospital of Medical School of Nanjing University (2016–057‐01) was obtained before setting out to conduct the research.

### 2.2 Cell transfection assay

Lentiviral shRNA, overexpression plasmid, and respective control for SNHG4, siRNA and overexpression plasmid for CREB5, and miR‐211‐5p mimics, miR‐211‐5p inhibitors, and their respective controls were synthesized and purified by RIBOBIO Gene (Guangzhou, Guangdong, China). Stable cell lines were generated by transduction in polybrene and harvested after puromycin (Sigma‐Aldrich, St. Louis, Missouri, USA) selection for 7 days. The above sequences were listed in the Table S3.

### 2.3 Bioinformatics analysis

The GEPIA database (http://gepia2.cancer‐pku.cn/) was analyzed to identify the relation between SNHG4 and pathological Stage of HCC, as well as the correlation between SNHG4 and the proliferating cell nuclear antigen. The HCC data in TCGA database was analyzed by R to reveal the expression of SNHG4 and the relation between SNHG4 and survival in HCC.

### 2.4 qRT‐PCR assay

In our study, the TRIzol was applied to extract the total RNA of HCC tissues and cells on the basis of the standard instructions. Then, the RNA was reversely transcribed by the PrimeScript™ RT Master Mix and SYBR® PrimeScript™ miRNA RT‐PCR Kit (TaKaRa, Shiga, Japan). The normalization of genetic level rests with U6 or β‐actin level, respectively. The primer sequences are displayed in Table S1.

### 2.5 Western blotting assay

The RIPA buffer (NCM Biotech) containing 1% protease inhibitor and 1% PMSF was applicable to extract the total protein. Tissues or cells were lysed for 30 min, during which the samples were vibrated every 10 min. After adding 5 × SDS‐PAGE Sample Loading Buffer, the lysis mixture was heated at 100°C. All protein samples were separated by 10% SDS‐PAGE and then transferred onto polyvinylidene fluoride (PVDF) membranes. The PVDF membranes were blocked by QuickBlock™ Blocking Buffer for Western Blot for 30 min. All antibodies were used in this study: CREB5 (Proteintech), E‐cadherin, Snail, Vimentin, and β‐actin antibodies and Secondary antibodies (Cell Signaling Technology).

### 2.6 Clone formation assay

The stable HCC cell lines (500 cells/well) were planted in the six‐well plates and cultured in the complete DMEM for 14 days. After rinsing with PBS and sequentially fixing with 4% formaldehyde for 10 min, the proliferating colonies (>50 cells/colony) were counted and dyed with crystal violet for 30 min.

### 2.7 Cell counting kit‐8 (CCK‐8) assay

The cellular proliferative ability was detected by CCK‐8 assay kit according to the manufacturers' protocols. The stable cells were planted in 96‐well plates (500 cells/well in 100 μl medium) and 10 μl CCK‐8 solution was added to each well. Once incubation for 2 h was completed, the absorbance (450 nm) of each well was observed.

### 2.8 Apoptosis assay

After harvesting the cells with trypsin without EDTA and rinsing with PBS for twice, we resuspended cells with 100 μl binding buffer which contained 5 μl Annexin V and 5 μl PI. Once the incubation for more than 10 min was complete, 400 μl binding buffer was used to resuspend the sample before detection. Data were obtained by a flow cytometer (BD Biosciences, San Jose, CA, USA) and analyzed by FlowJo software.

### 2.9 5‐Ethynyl‐2′‐deoxyuridine (EdU) assay

We seeded the stable cells into 96‐well plates (5000 cells/well) which embracing 100 μl medium. Once the 24 h‐incubation was finished, cells were incubated with EdU (50 μM) for 2 h at 37 °C. The cells were fixed with 4% paraformaldehyde for 30 min, and then permeabilized with 0.5% Triton X‐100. After performing EdU staining with Apollo dyeing reaction reagent for 30 min, we used Hoechest33342 to visualize the nuclei Finally, we observed the EdU positive cells under a fluorescence microscope.

### 2.10 Transwell assays

The cellular migratory and invasive abilities were detected through Transwell assay. HCC cells were planted in the upper chamber which contained 200 μl serum‐free medium, and coated with Matrigel (BD Biosciences) for invasion assays, and without Matrigel for migration assays. Meanwhile, the lower chamber was filled with 500 μl complete medium which acted as an attractant. After incubation for 24 h, the migratory or invasive cells were dyed with crystal violet for 30 min. After rinsing the chamber with PBS, we observed and obtained the stained cells through a light microscope.

### 2.11 Animal experiments

For exploring the tumor growth in vivo, we performed the subcutaneous tumorigenicity assay. HCC cells (10^6^ cells/100 μl per flank) were injected subcutaneously in BALB/c nude mice. Bidimensional tumor measurements were recorded with calipers every 4 days. After 20 days, the mice were euthanized, and the xenografts were weighed. All procedures involving animals were full of humanistic care and were approved by the Institutional Ethics Committee of the Affiliated Drum Tower Hospital of Nanjing University Medical School before the research began.

### 2.12 Subcellular fractionation

The nuclear and cytoplasmic fractions were extracted using the PARIS Kit (Invitrogen, USA) based on the manufacturer's protocol. U6 and β‐actin were used as the nuclear and cytosolic controls, respectively.

### 2.13 Dual‐luciferase reporter assay

Luciferase reporter vectors (pmirGLO) containing the mutant and wild‐type SNHG4 or CREB5 of the potential binding sites were cloned into a luciferase vector (Promega, Madison, WI, USA) and co‐transfected with miR‐211‐5p mimics into HCC cells. After incubation for 2 days, the luciferase activity was measured using a dual‐luciferase reporter assay system kit (Promega, USA) based on the manufacturer's instruction.

### 2.14 Fluorescent in situ hybridization

We performed the RNA FISH by means of a FISH kit (RiboBio, China) according to the manufacturer's instrctions. The DIG‐labeled SNHG4 probe was synthesized by Servicebio (Wuhan, China). We seeded 1 × 10^4^ HCC cells onto the slides which was placed in the 6‐well plates. After incubation overnight, the cells, undergoing fixing, permeabilizing and blocking, were mixed 20 μm probe mixture to hybridize with the target sequence. The next day, we obtained images via using a fluorescence microscope (Olympus FV1000, Japan).

### 2.15 Statistical analysis

All Data were displayed as mean ± SD. GraphPad Prism 8.0 were applied to analyze the data and figure the *p*‐value. The differences between multiple groups were evaluated via Student's *t*‐test. *p*‐value <0.05 was deemed statistically significant.

## 3 RESULTS

### 3.1 SNHG4 is upregulated in HCC and predicts a poor prognosis

**FIGURE 1:**
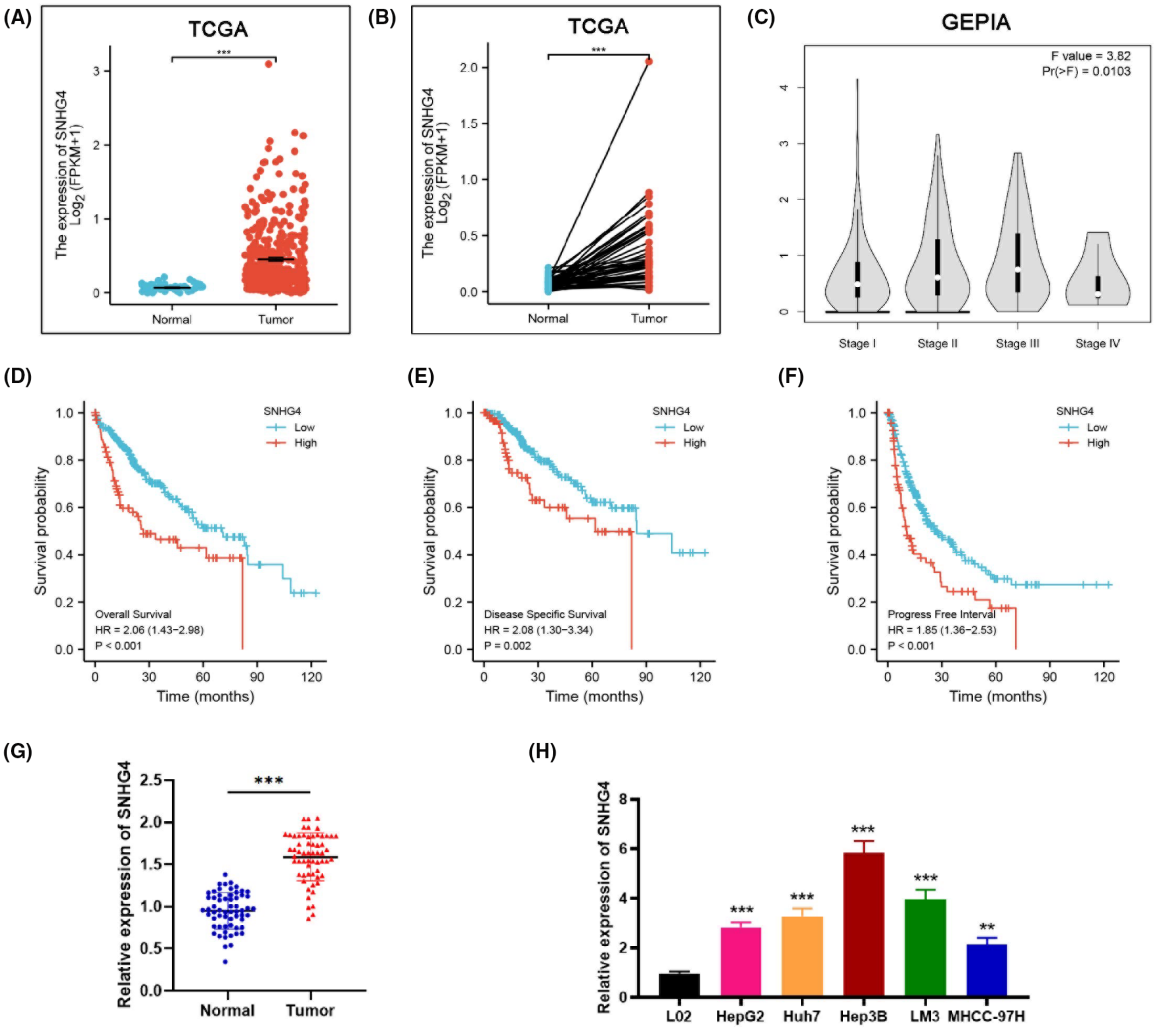
SNHG4 is upregulated in HCC and is associated with poor prognosis (A) TCGA data analysis of the expression of SNHG4 in HCC tissues (*n* = 374) and normal tissues (*n* = 50). (B) TCGA data analysis of the expression of SNHG4 in 50 paired HCC tumor and normal tissues. (C) Correlation between SNHG4 expression and HCC stage was analyzed through the GEPIA database. (D, E and F) Kaplan–Meier analysis of the relationship of SNHG4 with overall survival (D) or disease specific survival (E) or progress free interval (F) in patients with HCC based on the TCGA database. (G) qRT‐PCR analysis of the level of SNHG4 in 60 HCC tissues we collected. (H) qRT‐PCR analysis of the level of SNHG4 in HCC cell lines (HepG2, Huh7, Hep3B, LM3, MHCC‐97H) and normal liver cell (L02). Data represent means ± SD of at least three independent experiments. **p < 0.01, and ***p < 0.001.

The data concerning hepatocellular carcinoma (HCC) from The Cancer Genome Atlas (TCGA) was analyzed to identify the expression level of SNHG4, and both unpaired and paired analysis results displayed that the expression of SNHG4 in HCC tissues was notably higher than that in normal tissues (Figure 1A, B). We discovered that the level of SNHG4 owned a positive correlation with the HCC stage via analyzing the data from GEPIA database (Figure 1C). Meanwhile, the survival analyses were performed based the data from TCGA, we found that HCC patients with higher SNHG4 expression were featured with worse overall survival, disease specific survival, and progress free interval (Figure 1D–F). In line with the TCGA analysis, our qRT‐PCR data revealed that SNHG4 was significantly elevated in 60 HCC tissues than that in corresponding adjacent nontumor tissues (Figure 1G). The correlation analysis of SNHG4 expression and clinicopathologic features of HCC patients displayed that higher SNHG4 expression were remarkably associated with larger tumor size, microvascular invasion, and higher TNM and Edmonson stages (Table S2). Additionally, the upregulation of SNHG4 was detected in HCC cell lines (Figure 1H). Collectively, the above data demonstrated that SNHG4 is involved in HCC progression and possibly functions as a prognostic marker.

### 3.2 SNHG4 enhanced the malignancy of HCC cells

**FIGURE 2:**
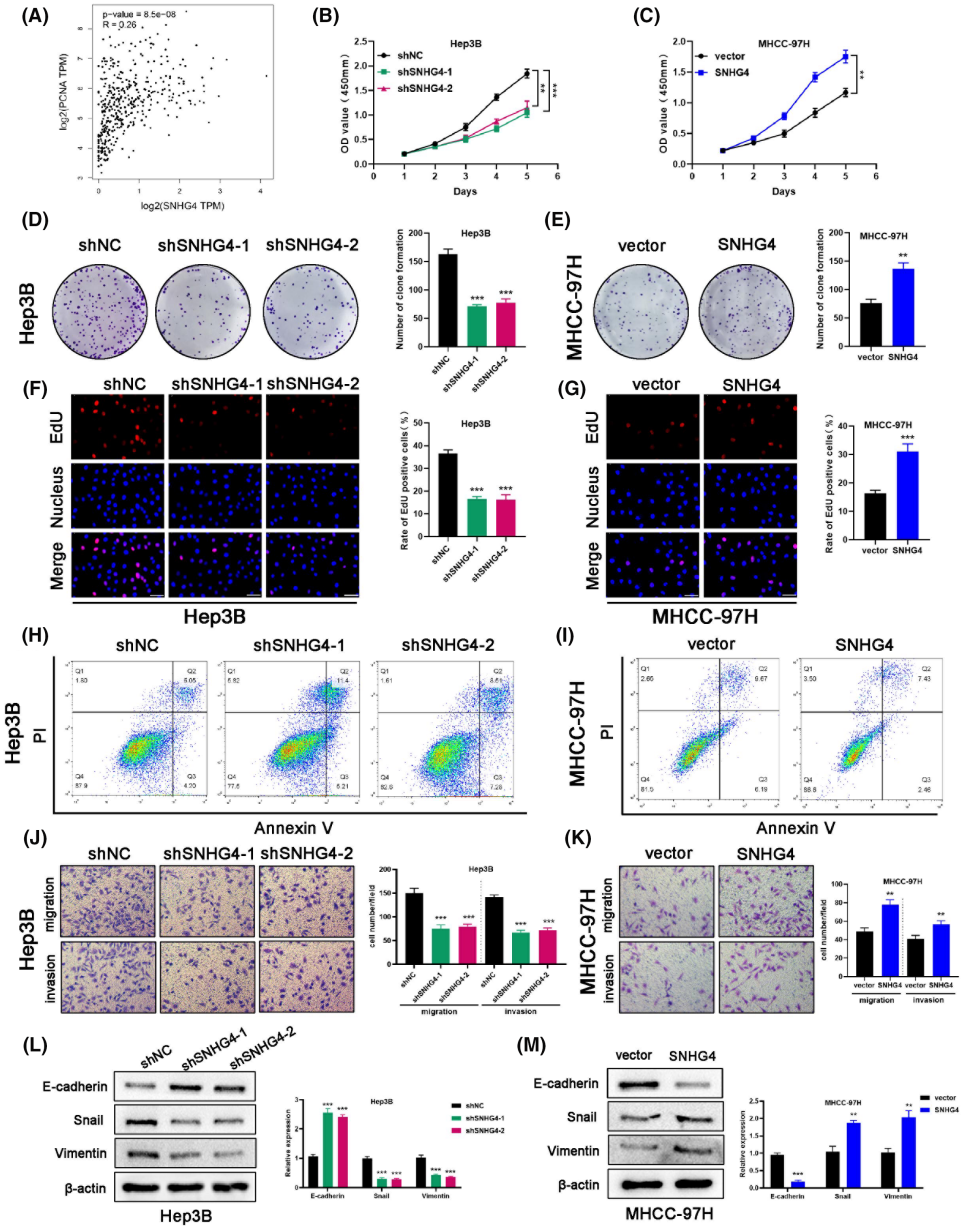
SNHG4 enhanced the malignancy of HCC cells. To explore the role of SNHG4 in malignancy of HCC, Hep3B was chosen for SNHG4 knockdown assays, and MHCC‐97 was chosen for SNHG4 overexpression assays. (A) Correlation between SNHG4 expression and PCNA was analyzed through the GEPIA database. (B and C) CCK‐8 assays evaluated the cell viability of in HCC cell lines with SNHG4 knockdown or overexpression. (D‐G) Colone formation and EdU staining assays evaluated the cellular proliferation in HCC cell lines with SNHG4 knockdown or overexpression. (H and I) Apoptosis level of HCC cell lines with SNHG4 knockdown or overexpression analyzed by flow cytometry. (J and K) Transwell assays detected the migration and invasion of HCC cell lines with SNHG4 knockdown or overexpression. (L and M) Western blotting analysis of E‐cadherin, Snail and Vimentin expression in HCC cell lines. Data represent means ± SD of at least three independent experiments. **p < 0.01, and ***p < 0.001.

For probing into the molecular function of SNHG4 in HCC, we constructed the SNHG4‐downregulated Hep3B cells and the SNHG4‐upregulated MHCC‐97H cells for functional experiments. The modulation efficiency of SNHG4 was examined via qRT‐PCR (Figure S1A,B). Then, a positive correlation between SNHG4 and the proliferating cell nuclear antigen (PCNA) was observed based on the data from GEPIA, which hinted us that SNHG4 may affect the proliferation of HCC (Figure 2A). The CCK8 assay results revealed that SNHG4 knockdown inhibited the proliferative ability of Hep3B cells (Figure 2B). In line with this, SNHG4 knockdown contributed to a remarkable decline in the clone formation of Hep3B cells (Figure 2D). The EdU incorporation assays results also demonstrated that SNHG4 knockdown reduced the proliferation of Hep3B cells from multiple perspectives (Figure 2F). Flow cytometry analysis uncovered that SNHG4 knockdown elevated the apoptotic level of Hep3B cells (Figure 2H and S1C). For the purpose of integrally elucidating the function of SNHG4, a series of functional experiments demonstrated that SNHG4 overexpression notably contributed to the enhanced proliferative capacities of MHCC‐97H cells (Figure 2C,E,G), as well as the inhibited apoptotic level (Figure 2I and S1D). Meanwhile, the transwell assays indicated that the migratory and invasive level of Hep3B cells were restricted once SNHG4 was downregulated (Figure 2J). As presented in the Figure 2K, SNHG4 overexpression accelerated the migratory and invasive level of MHCC‐97H cells. As has been widely reported, epithelial‐mesenchymal transition (EMT) participates in the progression of HCC.^21^, ^22^ Especially, it was reported that SNHG4 enhanced the EMT process through sponging miR‐204‐5p in gastric cancer.^23^ In order to explore whether SNHG4 enhanced migration and invasion via EMT in HCC, the EMT‐related markers were detected by WB assays. The data displayed that Hep3B cells with SNHG4 knockdown were characterized with elevated E‐cadherin expression and depressed Snail and Vimentin expression (Figure 2L). As expected, the opposite results were detected in MHCC‐97H cells with SNHG4 overexpression (Figure 2M). Moreover, we constructed the SNHG4‐upregulated Hep3B cells and the SNHG4‐downregulated MHCC‐97H cells for functional experiments (Figure S2). The CCK8 assay results revealed that SNHG4 overexpression promoted the cell viability of Hep3B cells (Figure S2A). In line with this, SNHG4 overexpression resulted in a remarkable increase in the clone formation and rate of EdU positive of Hep3B cells (Figure S2C, E). The transwell assay revealed that SNHG4 overexpression accelerated the migratory and invasive level of Hep3B cells (Figure S2G). Meanwhile, SNHG4 knockdown notably led to the inhibited viability and proliferative capacities of MHCC‐97H cells (Figure S2B,D,F), as well as the reduced migration and invasion (Figure S2H). Collectively, the above findings indicated SNHG4 promoted the malignancy of HCC cells.

### 3.3 SNHG4 promoted HCC tumor growth in vivo

**FIGURE 3:**
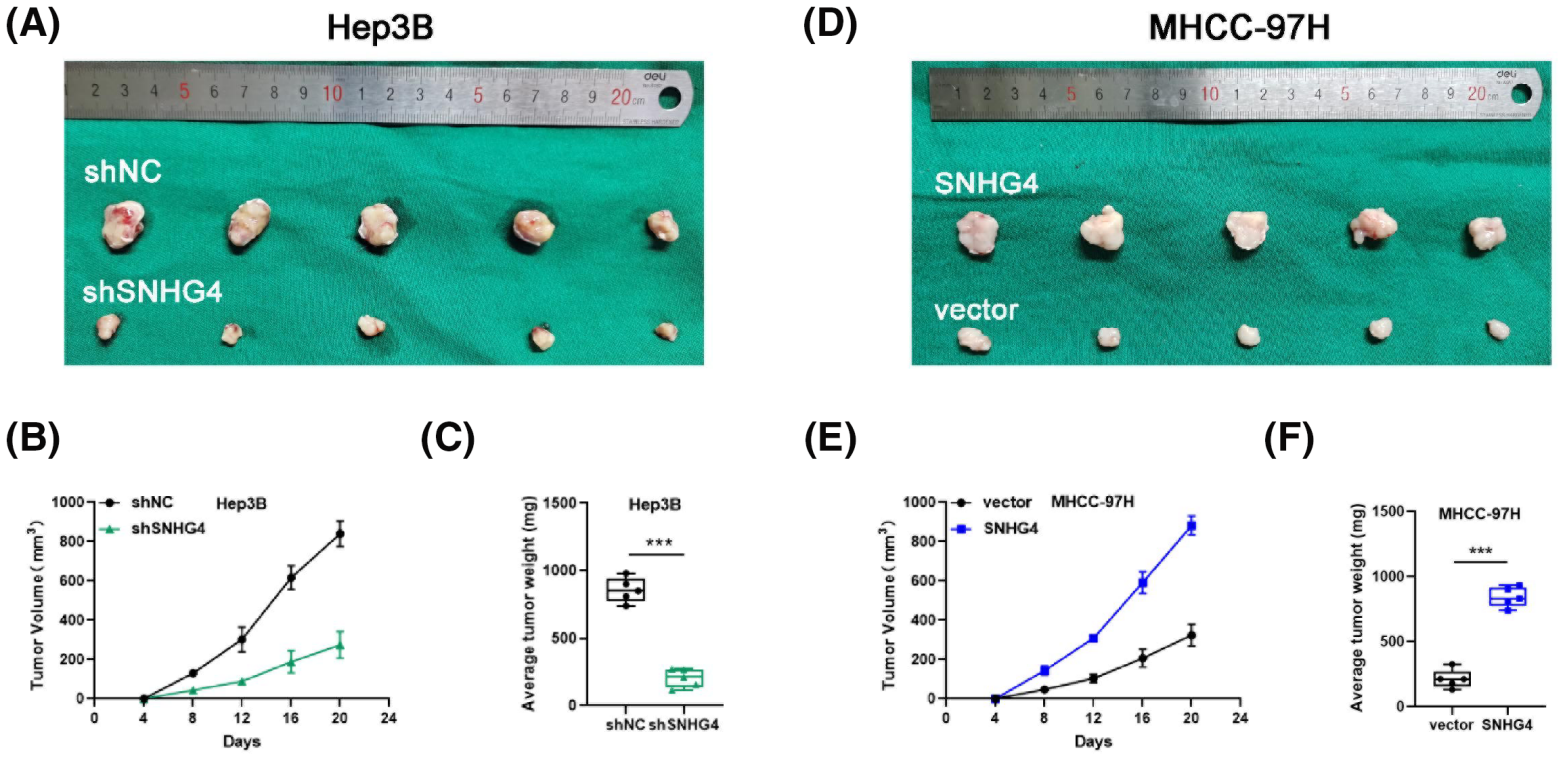
SNHG4 promoted HCC tumor growth in vivo (A and D) Representative images of xenograft HCC tumors obtained from mice inoculated with SNHG4‐downregulated Hep3B (shSNHG4) and its control Hep3B (shNC) or SNHG4‐upregulated MHCC‐97H (SNHG4) and its control MHCC‐97H (vector). (B and E) Growth curves for the tumor volumes were calculated for each group. (C and F) Tumor weight of xenograft HCC tumors in each group. Data represent means ± SD of at least three independent experiments. ***p < 0.001.

To ascertain whether SNHG4 was involved with tumor growth in vivo, Hep3B cells transfected with shNC or shSNHG4 were injected subcutaneously into nude mice. The data displayed that the slower growth and lower average tumor volume and weight were observed in the SNHG4‐downregulated Hep3B cells (Figure 3A–C). At the meantime, we found that the subcutaneous tumors from the SNHG4‐upregulated MHCC‐97H cells were characterized by accelerated growth and larger average tumor volume and weight (Figure 3D–F). Overall, the data suggested that SNHG4 accelerated the HCC tumor growth in vivo.

### 3.4 SNHG4 functions as a ceRNA and competitively binds miR‐211‐5p in HCC

**FIGURE 4:**
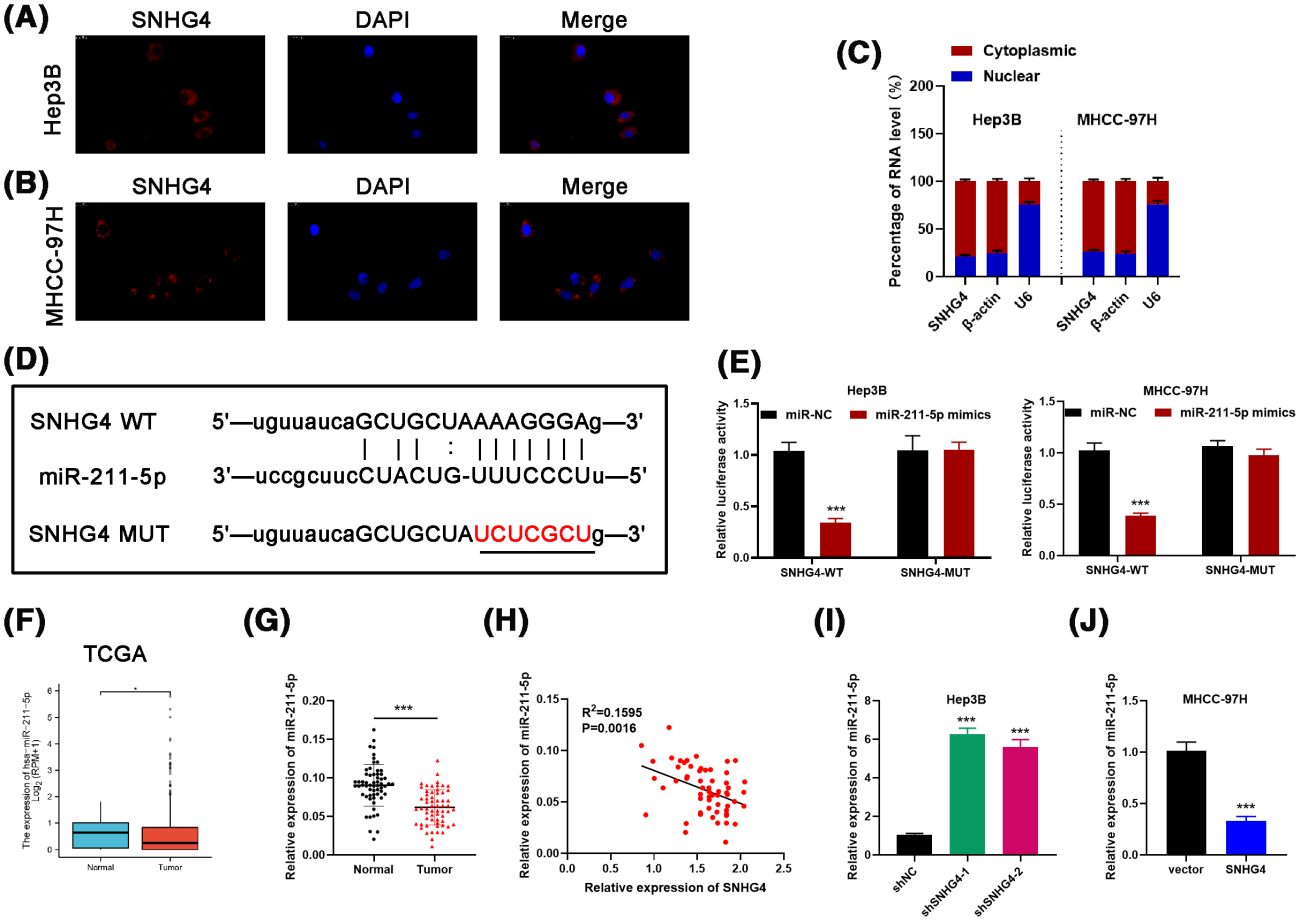
SNHG4 functions as a ceRNA and competitively binds miR‐211‐5p in HCC (A, B and C) FISH and qRT‐PCR analysis of the subcellular localization of SNHG4 in Hep3B and MHCC‐97H cell. (D, E) The dual‐luciferase reporter assay indicated that the relative luciferase activity of the wild‐type SNHG4 was reduced by miR‐211‐5p in Hep3B and MHCC‐97H cell. (F) Relative expression of miR‐211‐5p in HCC tissues based on TCGA database. (G) qRT‐PCR analysis of the relative level of miR‐211‐5p in 60 HCC tissues we collected. (H) The correlation between the expression of SNHG4 and miR‐211‐5p was analyzed through qRT‐PCR analysis in 60 HCC tissues. (I and J) qRT‐PCR analysis of miR‐211‐5p expression in Hep3B and MHCC‐97H cell. Data represent means ± SD of at least three independent experiments. *p < 0.05 and ***p < 0.001.

For digging deeper for the potential mechanism of SNHG4 in HCC, the subcellular localization of SNHG4 was detected via FISH and subcellular fractionation analysis which uncovered that SNHG4 was predominately located in the cytoplasm of HCC cells (Figure 4A–C). Subsequently, we performed the analysis via Starbase (http://starbase.sysu.edu.cn) and RNAinter (http://www.rnainter.org/) to seek the potential miRNA which interacted with SNHG4.^24^, ^25^ There were 21 overlapping miRNAs between the prediction results from two above websites (Figure S3A). Then, 21 miRNAs were analyzed to evaluate their respective expression pattern in HCC based on the data from TCGA. The data displayed that the expression of let‐7c‐5p, let‐7a‐5p, let‐7 g‐5p, let‐7b‐5p, miR‐377‐3p, miR‐495‐3p, miR‐204‐5p and miR‐211‐5p was downregulated in HCC tumor tissues (Figure S3B). The correlation between the above 8 miRNAs and SNHG4 were analyzed via Starbase, which suggested let‐7c‐5p and miR‐211‐5p respectively owned a negative correlation with SNHG4 (Figure S3C). Moreover, qRT–PCR analysis indicated that miR‐211‐5p might be the downstream molecule of SNHG4 (Figure S3D).

In order to determine whether SNHG4 directly binds to miR‐211‐5p, we carried out the dual‐luciferase reporter assay which revealed that miR‐211‐5p mimics merely caused a notable decline of the relative luciferase activity in SNHG4‐WT group. Meanwhile, miR‐211‐5p mimics failed to reduce the relative luciferase activity in SNHG4‐MUT group (Figure 4D,E). Next, the downregulation of miR‐211‐5p was observed by analyzing the data from TCGA and 60 HCC samples, which indicated that miR‐211‐5p might serve as the tumor‐suppressor in HCC (Figure 4F,G). Moreover, the negative correlation between SNHG4 and miR‐211‐5p was discovered in HCC (Figure 4H). With the downregulation of SNHG4, the level of miR‐211‐5p was significantly increased, while with the upregulation of SNHG4, the level of miR‐211‐5p was notably decreased (Figure 4I,J). Altogether, SNHG4 could bind onto miR‐211‐5p as a sponge and was negatively correlated with miR‐211‐5p in HCC.

### 3.5 SNHG4 promoted the malignancy of HCC cells via regulating miR‐211‐5p

**FIGURE 5:**
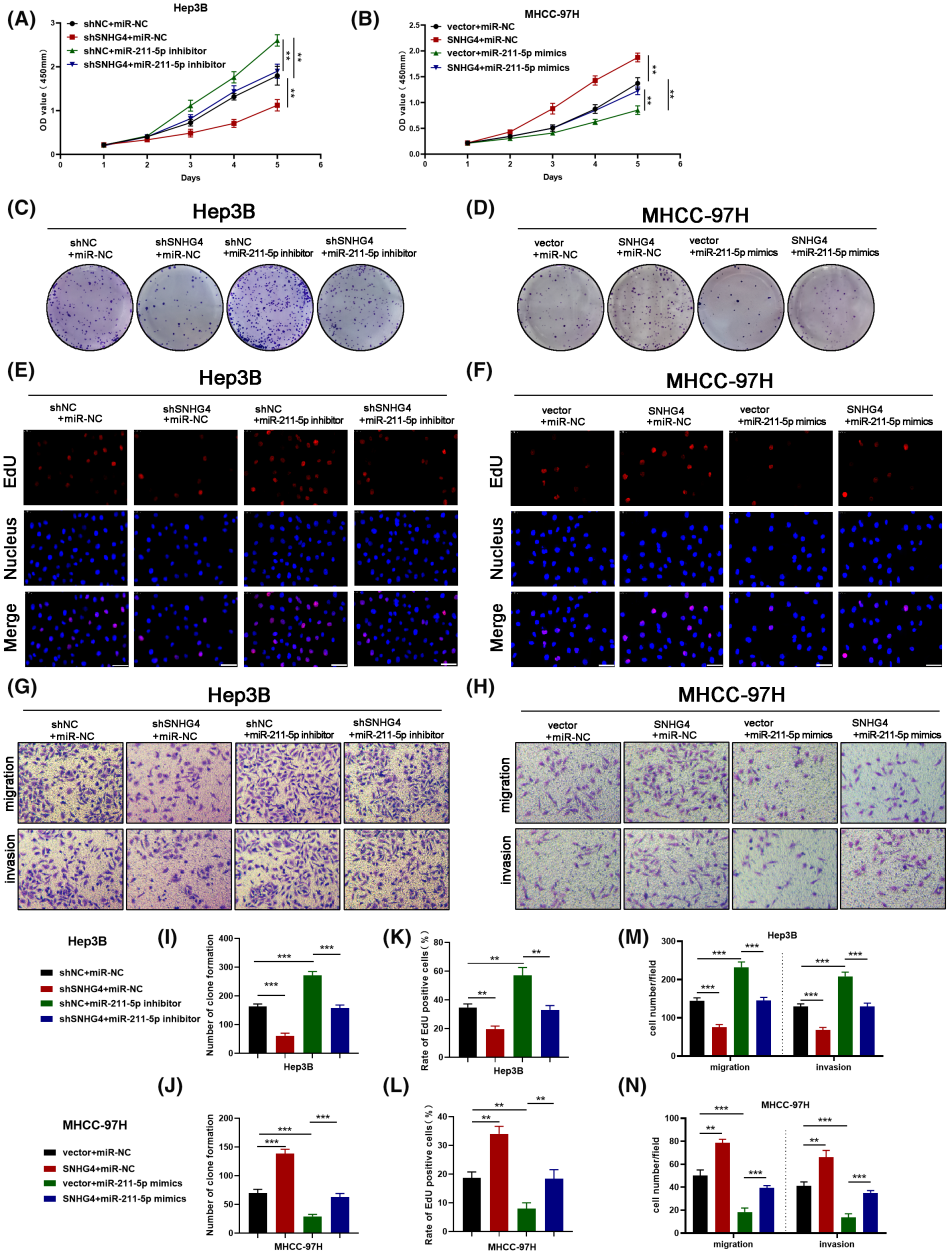
SNHG4 promoted the malignancy of HCC cells via regulating miR‐211‐5p (A and B) CCK‐8 assays evaluated the cell viability of in HCC cell lines from each group. (C‐F and I‐L) Colone formation and EdU staining assays evaluated the cellular proliferation in HCC cell lines from each group and the quantification. (G, H and M, N) Migration and invasion of HCC cells from each group were detected by Transwell assays and the quantification. Data represent means ± SD of at least three independent experiments. **p < 0.01, and ***p < 0.001.

To further probe into whether the oncogenic role of SNHG4 in HCC was dependent on miR‐211‐5p, we knocked down miR‐211‐5p in SNHG4‐downregulated Hep3B cells with miR‐211‐5p inhibitor, and upregulated miR‐211‐5p in SNHG4‐overexpressed MHCC‐97H cells with miR‐211‐5p mimics. The CCK8 assays uncovered that the repressed proliferation caused by SNHG4 knockdown could be partially rescued by silencing miR‐211‐5p in Hep3B cells (Figure 5A). Meanwhile, the accelerated proliferation caused by SNHG4 overexpression could be partially rescued by upregulating miR‐211‐5p in MHCC‐97H cells (Figure 5B). The reduced number of clone formation and EdU‐positive rate of SNHG4‐downregulated Hep3B cells were rescued by silencing miR‐211‐5p (Figure 5C,I,E,K). And the elevated number of clone formation and EdU‐positive rate of SNHG4‐upregulated MHCC‐97H cells were rescued by upregulating miR‐211‐5p (Figure 5D,J,F,L). At the meantime, miR‐211‐5p inhibition rescued the restrained migratory and invasive capacities of SNHG4‐downregulated Hep3B cells (Figure 5G,M). In addition, miR‐211‐5p overexpression rescued the enhanced migratory and invasive capacities of SNHG4‐overexpressed MHCC‐97H cells (Figure 5H,N). Collectively, SNHG4 facilitated the malignancy of HCC cells via negatively regulating miR‐211‐5p.

### 3.6 SNHG4 modulates CREB5 expression by competitively binding miR‐211‐5p

**FIGURE 6:**
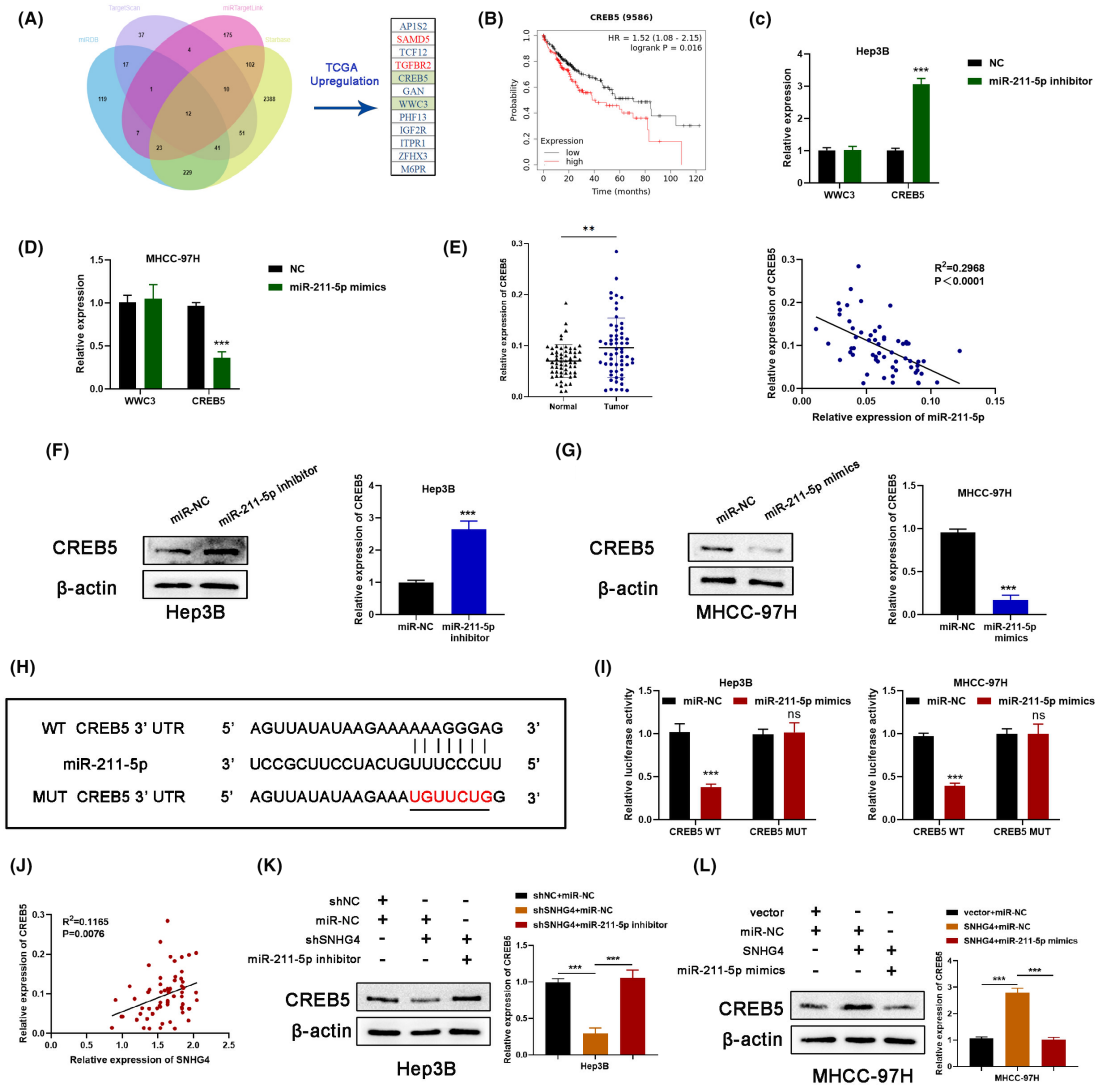
SNHG4 modulates CREB5 expression by competitively binding miR‐211‐5p (A) Venn diagram displaying the potential downstream targets of miR‐211‐5p. (B) Overall survival analysis based on CREB5 expression in HCC patients from TCGA database. (C and D) qRT‐PCR analysis of the level of WWC3 and CREB5 after miR‐211‐5p silencing in Hep3B and MHCC‐97H cell. (E) qRT‐PCR analysis of the level of CREB5 and the correlation between the expression of CREB5 and miR‐211‐5p was analyzed in 60 HCC tissues we collected. (F and G) Western blotting analysis of CREB5 expression when knockdown or overexpression of miR‐211‐5p in HCC cells. (H and I) The dual‐luciferase reporter assay indicated that the relative luciferase activity of the wild‐type CREB5 was reduced by miR‐211‐5p. (J) The correlation between the expression of CREB5 and SNHG4 was analyzed through qRT‐PCR analysis in 60 HCC tissues we collected. (K and L) Western blotting analysis of CREB5 expression after transfection in HCC cells. Data represent means ± SD of at least three independent experiments. ***p < 0.001.

In order to determine the targets of miR‐211‐5p, the databases containing miRDB (http://mirdb.org/mirdb), TargetScan (http://www.targetscan.org), miRTargetLink (www.ccbweb.cs.uni‐saarland.de/mirtargetlink/), and Starbase (http://starbase.sysu.edu.cn) were analyzed. Finally, 12 overlapping potential target genes were observed in four databases (Figure 6A).^24^, ^26^, ^27^, ^28^ We screened 10 upregulated genes in HCC via analyzing TCGA database (Figure S4A). Next, the survival analysis results showed that high expression of CREB5 and WWC3 were associated with shorter overall survival in HCC patients, respectively (Figure 6B and Figure S4B). The qRT‐PCR analysis uncovered that CREB5 expression was notably upregulated in Hep3B cells with miR‐211‐5p inhibition (Figure 6C), and CREB5 expression was notably restrained in MHCC‐97H cells with miR‐211‐5p overexpression (Figure 6D). Meanwhile, the negative correlation between CREB5 and miR‐211‐5p in HCC was screened based on TCGA data (Figure S4C). All above findings suggested that CREB5 may be the target of miR‐211‐5p. Further experiments demonstrated that the upregulation of CREB5 and the negative correlation between miR‐211‐5p and CREB5 were detected in HCC tumor tissues (Figure 6E). Then, western blot analysis revealed that CREB5 expression was increased in Hep3B cells with miR‐211‐5p inhibitor transfection, but reduced in MHCC‐97H cells with miR‐211‐5p mimics transfection (Figure 6F,G). The subsequent dual‐luciferase reporter assay indicated that miR‐211‐5p mimics merely resulted in a notable decline of the relative luciferase activity in CREB5‐WT group. Meanwhile, miR‐211‐5p mimics failed to reduce the relative luciferase activity in CREB5‐MUT group (Figure 6H,I). The positive correlation between SNHG4 and CREB5 were detected in HCC tumor tissues we collected (Figure 6J). In Hep3B cells, the declined protein level of CREB5 caused by SNHG4 knockdown was partially offset by co‐transfection with the miR‐211‐5p inhibitor. The opposite results were detected in SNHG4‐overexpressed MHCC‐97H with miR‐211‐5p mimics transfection (Figure 6K,L). Collectively, our findings displayed SNHG4 modulated CREB5 expression via competitively binding miR‐211‐5p.

### 3.7 Effects of SNHG4 on the malignancy of HCC cells could be reversed by CREB5

**FIGURE 7:**
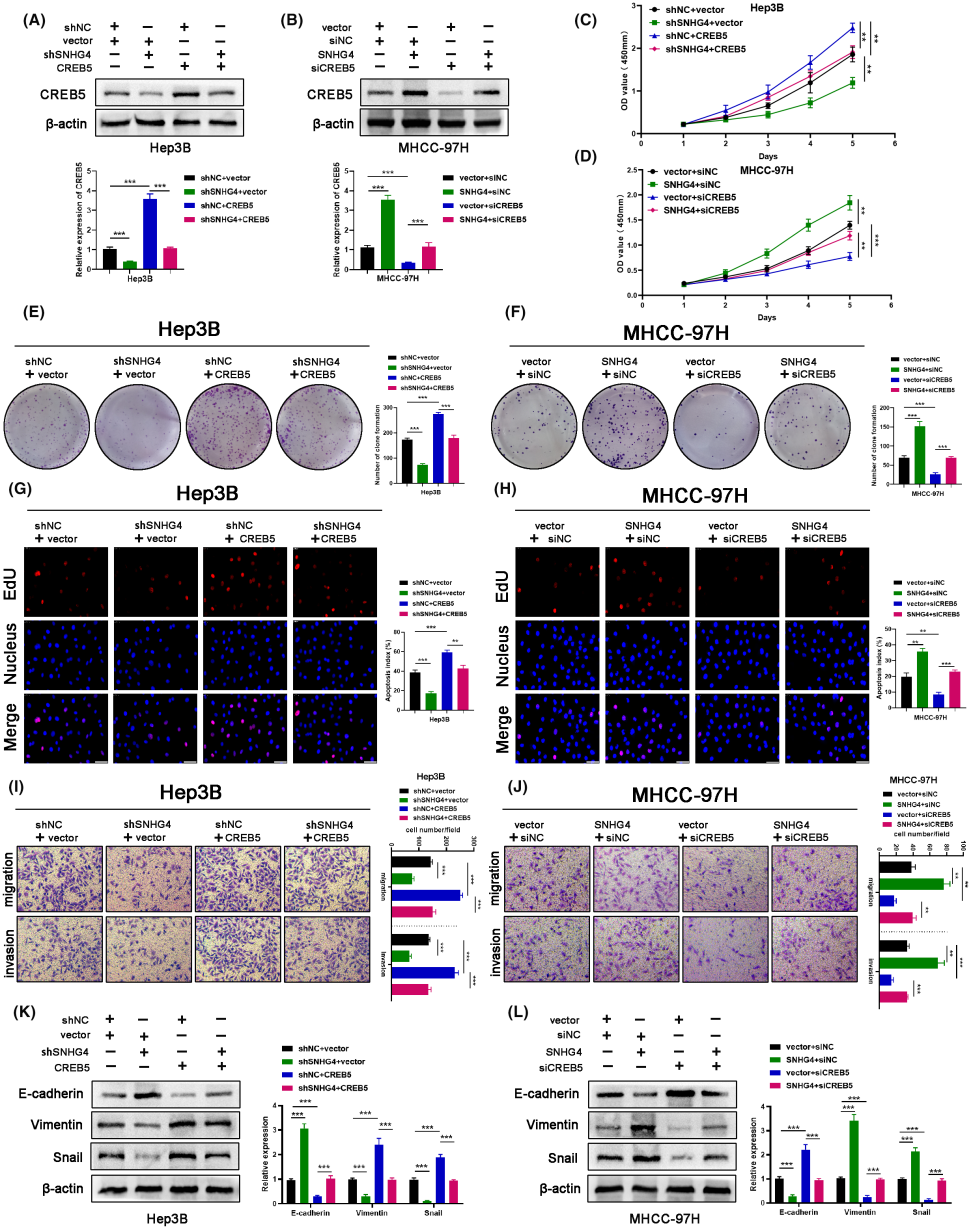
Effects of SNHG4 on the malignancy of HCC cells could be reversed by CREB5 (A and B) Western blotting analysis of CREB5 expression after transfection in Hep3B and MHCC‐97H cells. (C and D) CCK‐8 assays evaluated the cell viability of in HCC cell lines from each group. (E and G) Cell proliferation in Hep3B cells from each group was evaluated through colone formation and EdU assays. (F and H) Cell proliferation in MHCC‐97H cells from each group was evaluated through colone formation and EdU assays. (I and J) Migration and invasion of HCC cells from each group were detected by Transwell assays. (K and L) Western blotting analysis of E‐cadherin, Vimentin and Snail expression in HCC cells from each group. Data represent means ± SD of at least three independent experiments. **p < 0.01, and ***p < 0.001.

To further identify whether the oncogenic role of SNHG4 in HCC relied on the interaction with CREB5, SNHG4‐downregulated Hep3B cells were co‐transfected with LV‐CREB5. Meanwhile, SNHG4‐overexpressed MHCC‐97H cells were co‐transfected with siCREB5. The transfection efficiency was detected via western blotting (Figure 7A,B). The CCK8 assay, clone formation and EdU staining results revealed that CREB5 overexpression could partially counteract the inhibited proliferation of Hep3B cells mediated by SNHG4 downregulation (Figure 7C,E,G). At the meantime, CREB5 downregulation could partially counteract the enhanced proliferation of MHCC‐97H cells mediated by SNHG4 upregulation (Figure 7D,F,H). The impact of SNHG4 knockdown on the cellular migration and invasion, and the expression of EMT‐related markers in Hep3B cells were offset by CREB5 overexpression (Figure 7I,K). Moreover, CREB5 knockdown rescued the enhanced migration and invasion, and the altered expression of EMT‐related markers of SNHG4‐overexpressed MHCC‐97H cells (Figure 7J,L). Altogether, we demonstrated that SNHG4 promoted HCC progression through modulating the expression of CREB5.

## 4 DISCUSSION

Hepatocellular carcinoma (HCC) has been identified as one of the most lethal and prevalent tumors, but the precise mechanism of the initiation and development remains vague.^29^, ^30^ Numerous studies focusing the irreplaceable role of ncRNA, peculiarly lncRNAs and miRNAs, in the diagnosis and treatment to HCC, emerged within the recent years.^31^ Currently, lncRNA SNHG4 has been reported to be involved in a variety of diseases.^32^ For instance, SNHG4 restrained cell apoptosis in diabetic retinopathy via sponging miR‐200b.^33^ SNHG4 inhibited METTL3‐mediated m6A level of STAT2 mRNA, which resulted in the alleviated LPS‐induced inflammatory lung injury.^34^ It was reported that SNHG4 promoted the progression of non‐small cell lung cancer (NSCLC) via modulating microRNA‐let‐7 e/KDM3A/p21 pathway.^18^ Downregulation of SNHG4 inhibited the proliferation, migration, invasiveness and EMT of NSCLC via regulating miR‐98‐5p.^35^ In prostate cancer, SNHG4 upregulated by SP1 accelerated the malignancy through upregulating ZIC5 expression via competitively binding miR‐377.^36^ In gastric cancer, SNHG4 enhanced the malignant behavior including proliferation, migration, invasion and EMT through sponging miR‐204‐5p.^23^ In colorectal cancer, SNHG4 promoted the cellular proliferation via accelerating cell cycle through regulating miR‐590‐3p/CDK1 axis.^37^ Nevertheless, SNHG4 was downregulated in acute myeloid leukemia cells, and inhibited the proliferation via modulating the miR‐10a/PTEN axis.^38^

Although SNHG4 has been reported to be highly expressed in liver cancer and associated with prognosis,^39^ the definite molecular functions of lncRNA SNHG4 in HCC remain uncertain. In our present study, TCGA data analysis and qRT‐PCR analysis revealed SNHG4 was upregulated in HCC. The survival analysis uncovered that SNHG4 overexpression was associated with worse prognosis, suggesting that SNHG4 may serve as an oncogenic role in HCC. The subsequent functional experiments displayed that SNHG4 enhanced the cellular proliferation, migration and invasion and accelerated the tumor growth in vivo.

The significance of subcellular localization for the biological function of lncRNAs had been highlighted.^40^, ^41^ Cytoplasmic lncRNAs possess the characteristics of being able to bind to miRNAs, thus affecting the stability or translation of mRNA, which are depicted as ceRNA networks.^42^, ^43^ For instance, LINC01612 regulated the ATF3/p53 axis through sponging miR‐494 to inhibit the development of HCC.^44^ lncRNA NEAT1 facilitated the expression of MIOX via competitively binding to miR‐362‐3p, leading to the enhanced ferroptosis in HCC cells.^11^ In this study, we discovered that SNHG4 was chiefly located in cytoplasm, so we hypothesized that SNHG4 acted as a ceRNA for the functional approach. Subsequently, through bioinformatics analysis and luciferase reporter assays, miR‐211‐5p was found to bind to SNHG4. It had been reported in previous studies that miR‐211‐5p functioned as a tumor suppressor in multiple tumors, such as HCC, colorectal cancer and NSCLC, and so on.^45^, ^46^, ^47^ Downregulation of miR‐211‐5p was detected in HCC, and the relative level of SNHG4 was negatively correlated with that of miR‐211‐5p. What's more, the functional experiments uncovered that SNHG4 facilitated the malignancy of HCC cells via negatively regulating miR‐211‐5p.

Competitive binding of lncRNA and miRNA would alter the level of miRNA target genes.^13^, ^48^ To explore the target gene of miR‐211‐5p, the bioinformatics analysis (miRDB, TargetScan, miRTargetLink and Starbase) and the luciferase reporter assays were performed, and cAMP responsive element binding protein 5 (CREB5) was identified as the direct target of miR‐211‐5p. It is reported that CREB5 is located on chromosome 7 (7p15.1).^49^ It had been reported that CREB5 facilitated multiple tumors progression, such as colorectal cancer and prostate cancer.^50^, ^51^ In addition, the ceRNA network of SNHG4/miR‐211‐5p/CREB5 in HCC has not been demonstrated before. Our findings revealed that CREB5 was upregulated in HCC and correlated with poor prognosis. The functional experiments displayed that the oncogenic role of SNHG4 could be modulated by miR‐211‐5p/CRRB5 axis.

In general, lncRNA SNHG4 enhanced the expression of CREB5 by sponging miR‐211‐5p, thus promoting the malignant behavior of HCC cells. Our findings indicated that SNHG4 could be a key tumor promoter and a potential therapeutic target for HCC.

## AUTHOR CONTRIBUTIONS

**Jiannan Qiu:** Formal analysis (lead); investigation (lead); writing – original draft (lead). **peng wang:** Data curation (equal); formal analysis (lead). **zheng chen:** Data curation (equal); formal analysis (equal). **yan zhou:** Data curation (equal). **guang zhang:** Data curation (equal); formal analysis (equal). **zhongxia wang:** Formal analysis (equal); investigation (equal). **junhua wu:** Conceptualization (equal); funding acquisition (equal); writing – review and editing (equal). **qiang zhu:** Conceptualization (equal); funding acquisition (equal); writing – review and editing (equal). **chunping jiang:** Conceptualization (lead); funding acquisition (equal); writing – review and editing (equal).

## FUNDING INFORMATION

The research was supported by Research Project of Jinan Microecological Biomedicine Shandong Laboratory (JNL‐202204A, JNL‐2022019B), Natural Science Foundation of Jiangsu Province (BK20190138), National Natural Science Foundation of China (81972888 and 81602093); the Primary Research & Development Plan of Jiangsu Province (BE2018701, BE2022840).

## CONFLICT OF INTEREST

All authors listed in our study have confirmed that they absolutely have no conflict of interest.

## Supporting information


Figure S1.

Figure S2.

Figure S3.

Figure S4.

Table S1.

Table S2.

Table S3.

## Data Availability

All data that obtained and analyzed during our study are available from the corresponding author once reasonably requested.
