# Supplementary figures and images for: Long noncoding RNA SNHG4 promotes the malignant progression of hepatocellular carcinoma through the miR‐211‐5p/CREB5 axis
Source: Cancer Med. 2022 Dec 23;12(7):8388–402. doi: 10.1002/cam4.5559 (PMC10134289; doi:10.1002/cam4.5559)

**A**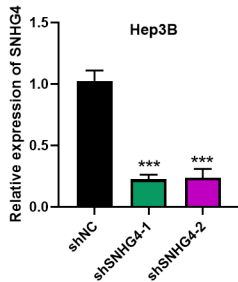**B**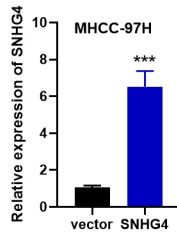**C**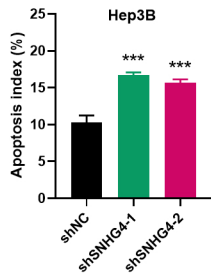**D**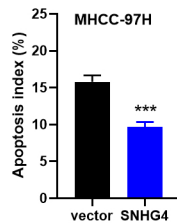

Supplement: Supplementary file 1 — Figure S1. [file CAM4-12-8388-s002.pdf]

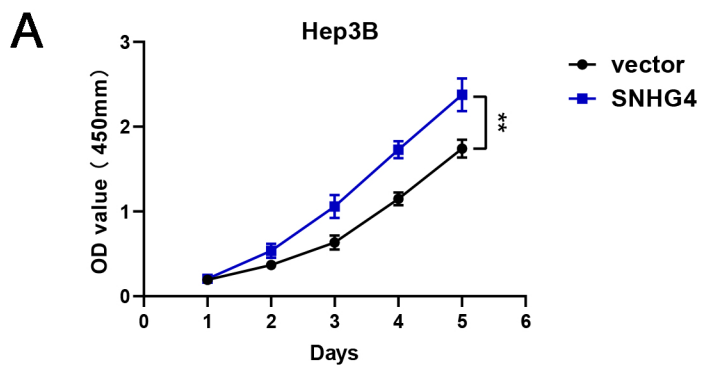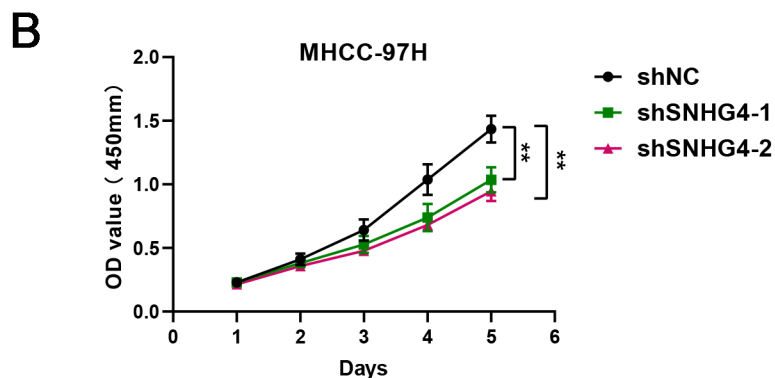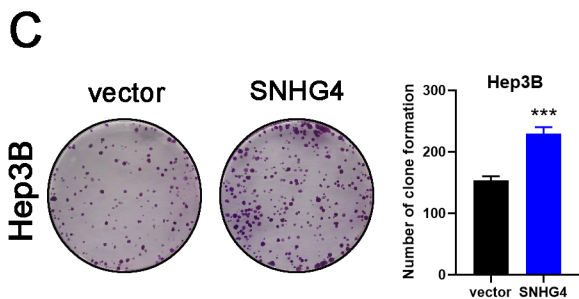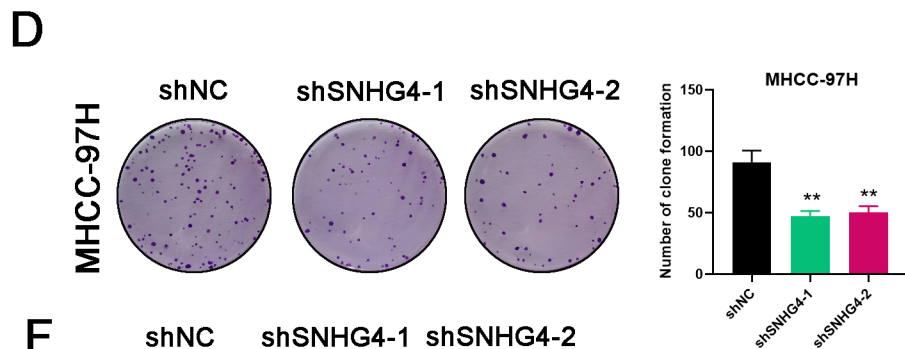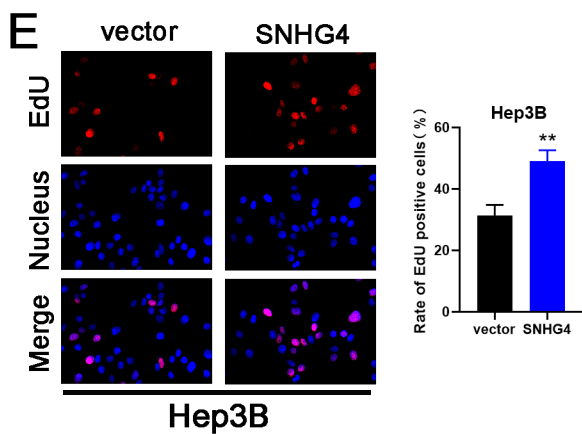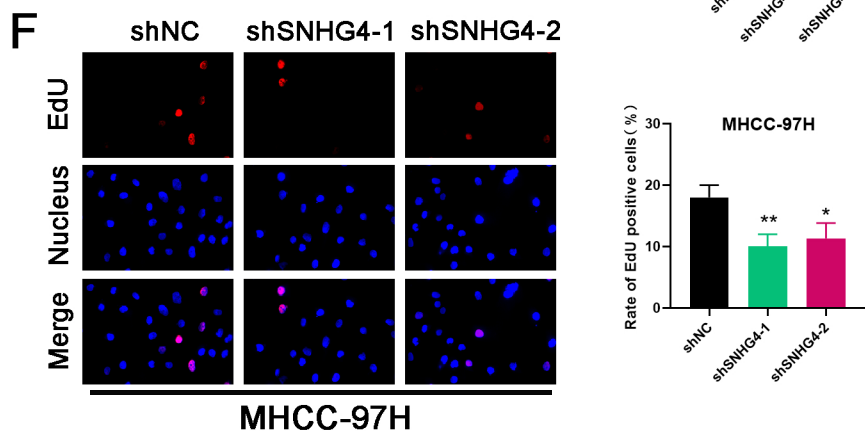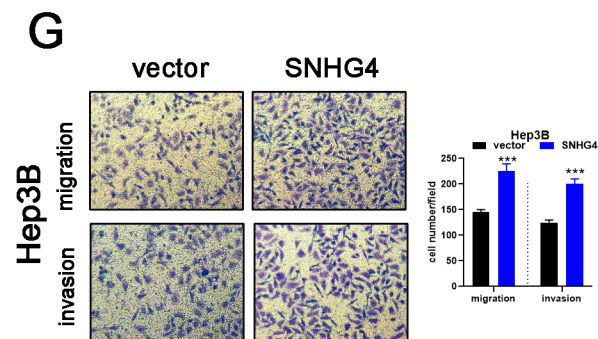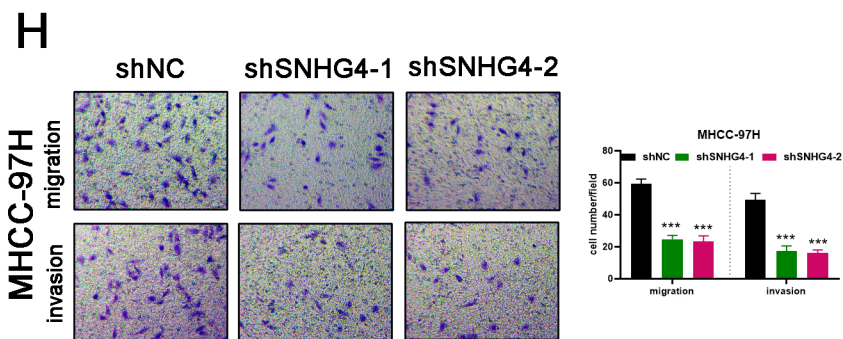

Supplement: Supplementary file 2 — Figure S2. [file CAM4-12-8388-s007.pdf]

A

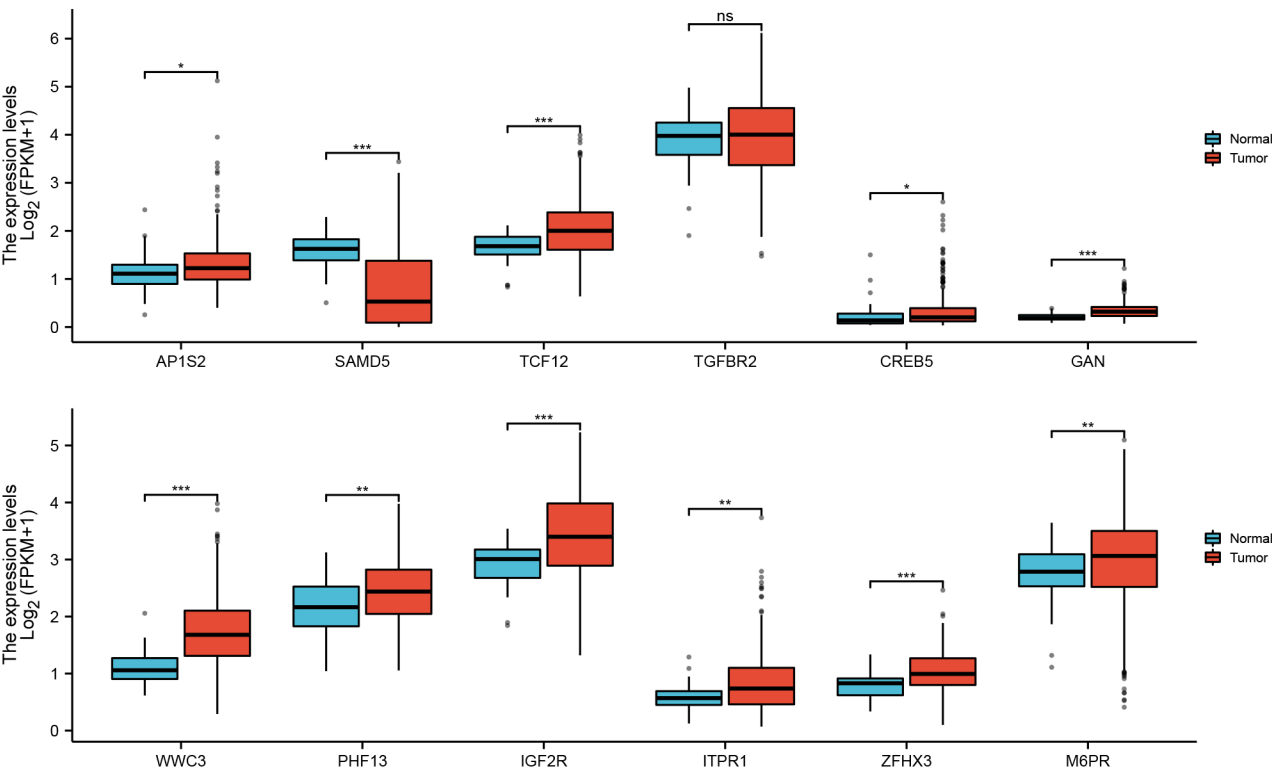

B

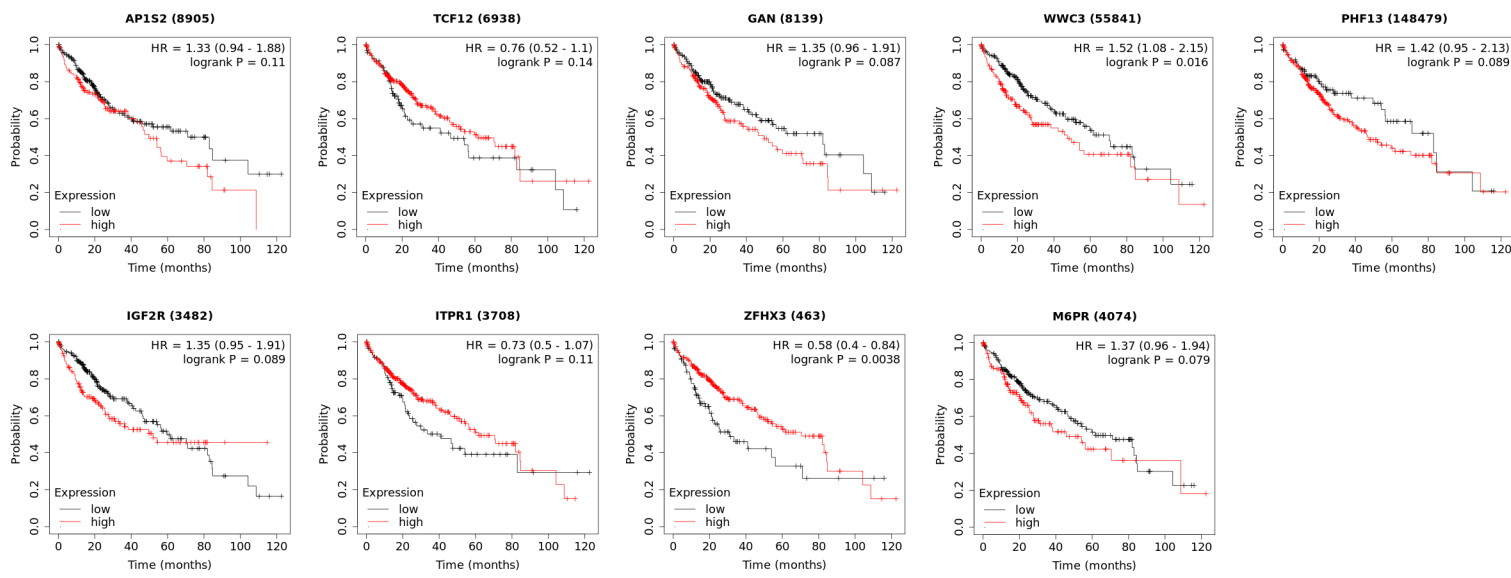

C

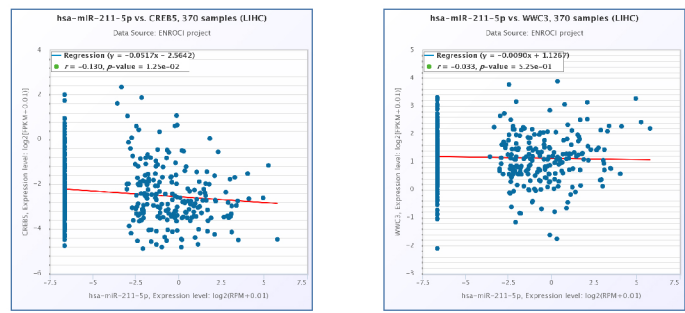

Supplement: Supplementary file 4 — Figure S4. [file CAM4-12-8388-s004.pdf]
