# Supplementary material for: Long noncoding RNA SNHG4 promotes the malignant progression of hepatocellular carcinoma through the miR‐211‐5p/CREB5 axis
Source: Cancer Med. 2022 Dec 23;12(7):8388–402. doi: 10.1002/cam4.5559 (PMC10134289; doi:10.1002/cam4.5559)
Supplement: Supplementary file 3 — Figure S3. [file CAM4-12-8388-s005.pdf]

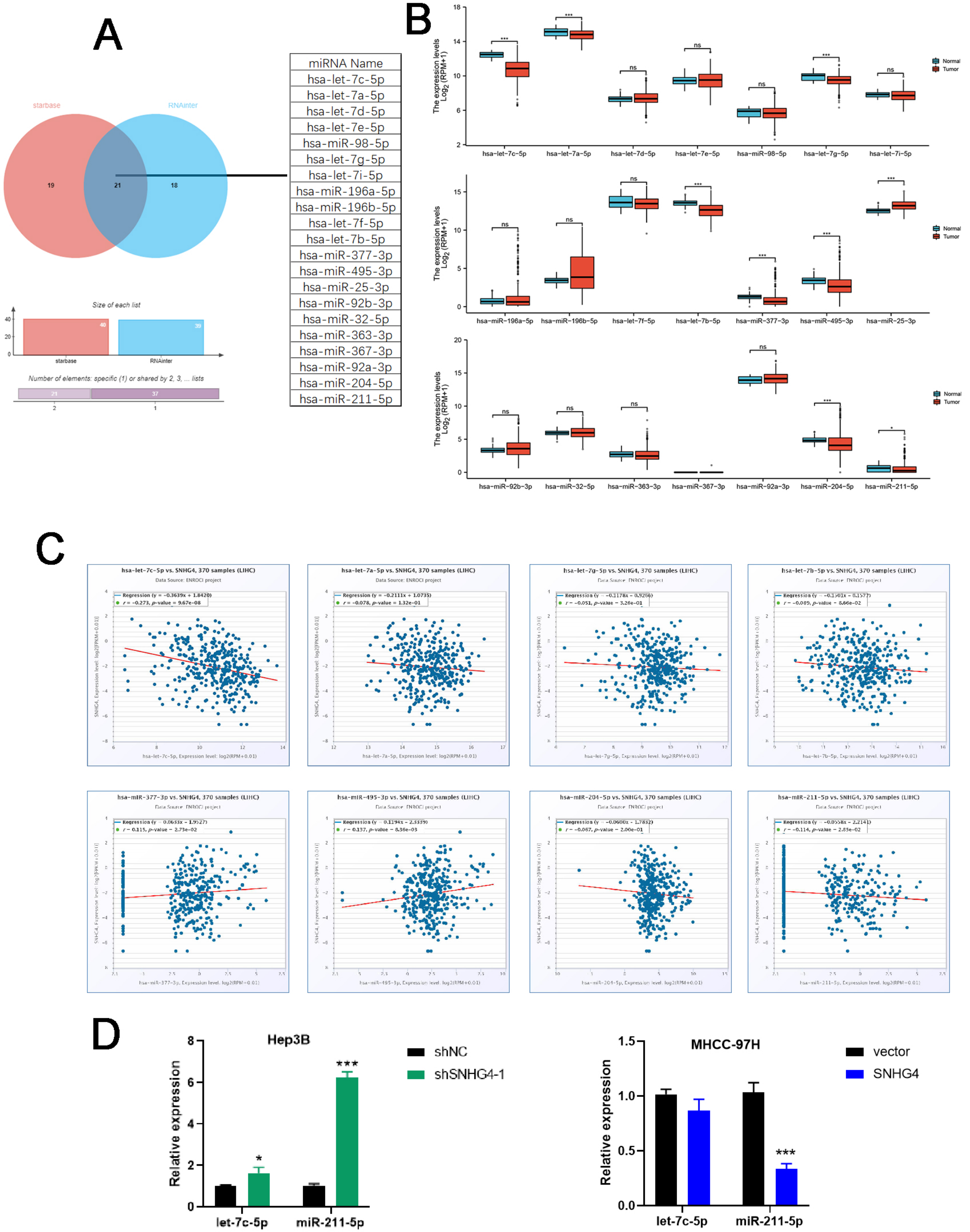
A
starbase
RNAinter
19
21
18
Size of each list
40
39
starbase
RNAinter
Number of elements: specific (1) or shared by 2, 3, ... lists
21
37
2
1
miRNA Name
hsa-let-7c-5p
hsa-let-7a-5p
hsa-let-7d-5p
hsa-let-7e-5p
hsa-miR-98-5p
hsa-let-7g-5p
hsa-let-7i-5p
hsa-miR-196a-5p
hsa-miR-196b-5p
hsa-let-7f-5p
hsa-let-7b-5p
hsa-miR-377-3p
hsa-miR-495-3p
hsa-miR-25-3p
hsa-miR-92b-3p
hsa-miR-32-5p
hsa-miR-363-3p
hsa-miR-367-3p
hsa-miR-92a-3p
hsa-miR-204-5p
hsa-miR-211-5p
B
The expression levels Log2 (RPM+1)
Normal
Tumor
hsa-let-7c-5p
hsa-let-7a-5p
hsa-let-7d-5p
hsa-let-7e-5p
hsa-miR-98-5p
hsa-let-7g-5p
hsa-let-7i-5p
hsa-miR-196a-5p
hsa-miR-196b-5p
hsa-let-7f-5p
hsa-let-7b-5p
hsa-miR-377-3p
hsa-miR-495-3p
hsa-miR-25-3p
hsa-miR-92b-3p
hsa-miR-32-5p
hsa-miR-363-3p
hsa-miR-367-3p
hsa-miR-92a-3p
hsa-miR-204-5p
hsa-miR-211-5p
C
hsa-let-7c-5p vs. SNHG4, 370 samples (LIHC)
Data Source: ENROCI project
Regression (y = -0.3639x + 1.8420)
r = -0.273, p-value = 9.67e-08
hsa-let-7a-5p vs. SNHG4, 370 samples (LIHC)
Regression (y = -0.2111x + 1.0735)
r = -0.078, p-value = 1.32e-01
hsa-let-7g-5p vs. SNHG4, 370 samples (LIHC)
hsa-let-7b-5p vs. SNHG4, 370 samples (LIHC)
hsa-miR-377-3p vs. SNHG4, 370 samples (LIHC)
hsa-miR-495-3p vs. SNHG4, 370 samples (LIHC)
hsa-miR-204-5p vs. SNHG4, 370 samples (LIHC)
hsa-miR-211-5p vs. SNHG4, 370 samples (LIHC)
D
Hep3B
Relative expression
shNC
shSNHG4-1
let-7c-5p
miR-211-5p
MHCC-97H
vector
SNHG4
