# Supplementary material for: Long noncoding RNA SNHG4 promotes the malignant progression of hepatocellular carcinoma through the miR‐211‐5p/CREB5 axis
Source: Cancer Med. 2022 Dec 23;12(7):8388–402. doi: 10.1002/cam4.5559 (PMC10134289; doi:10.1002/cam4.5559)
Supplement: Supplementary file 5 — Table S1. [file CAM4-12-8388-s006.docx]

**Supplementary table 1: Primer sequences for the amplification**.

| Gene | Forward Primer (5′ → 3′) | Reverse Primer (5′ → 3′) |
| --- | --- | --- |
| SNHG4 | GCAGGTGACAGTCTGCATGT | TTTTAAGTCCCCTACCCCCATC |
| β-actin | GGCACCCAGCACAATGAA | CGGACTCGTCATACTCCTGCT |
| miR-211-5p | GATGCTGTAATGGATGATATGA | ATTGGAACGATACAGAGAAGATT |
| U6 | AAATCGCGCTCAGCGACGATC | CAACCATCGACTACGAGCT |
| CREB | CCCTGCCCAACCCTACAATG | GGACCTTGCATCCCCATGAT |
| WWC3 | CAAGAGCGCATGTTGAAGGAA | CGCTGCTGCTTAATCTGGTAGA |
