# Supplementary material for: Long noncoding RNA SNHG4 promotes the malignant progression of hepatocellular carcinoma through the miR‐211‐5p/CREB5 axis
Source: Cancer Med. 2022 Dec 23;12(7):8388–402. doi: 10.1002/cam4.5559 (PMC10134289; doi:10.1002/cam4.5559)
Supplement: Supplementary file 6 — Table S2. [file CAM4-12-8388-s001.docx]

**Table 2.** Correlation between SNHG4 expression and clinicopathological features in patients with HCC (n = 60).

|  | | | | |
| --- | --- | --- | --- | --- |
| Characteristics | Number | SNHG4 expression | | P-value |
|  |  | Low group | High group |  |
| Age(years) |  |  |  |  |
| <50 | 19 | 9 | 10 | 0.781 |
| ≥50 | 41 | 21 | 20 |  |
| Gender |  |  |  |  |
| FeMale | 15 | 8 | 7 | 0.766 |
| Male | 45 | 22 | 23 |  |
| Cirrhosis  Present  Absent  HBV infection  Positive  Negative  Tumor size(cm) | 43  17  43  17 | 19  11  21  9 | 24  6  22  8 | 0.152  0.775 |
| <5 | 24 | 16 | 8 | 0.035* |
| ≥5 | 36 | 14 | 22 |  |
| Microvascular invasion  Presence  Absence | 27  33 | 6  24 | 21  9 | <0.001*** |
| Tumor multiplicity  Simple  Multiple | 29  31 | 17  13 | 12  18 | 0.197 |
| α-fetoprotein (ng/ml)  ≤20  >20  TNM stage | 25  35 | 11  19 | 14  16 | 0.432 |
| I | 34 | 22 | 12 | 0.009** |
| II/III | 26 | 8 | 18 |  |
| Edmonson stage |  |  |  |  |
| I/II | 39 | 26 | 13 | <0.001*** |
| III/IV | 21 | 4 | 17 |  |

| *P<0.05, **P<0.01, ***P<0.001 |
| --- |
