# Supplementary material for: Long noncoding RNA SNHG4 promotes the malignant progression of hepatocellular carcinoma through the miR‐211‐5p/CREB5 axis
Source: Cancer Med. 2022 Dec 23;12(7):8388–402. doi: 10.1002/cam4.5559 (PMC10134289; doi:10.1002/cam4.5559)
Supplement: Supplementary file 7 — Table S3. [file CAM4-12-8388-s003.docx]

**Long noncoding RNA SNHG4 promotes the malignant progression of hepatocellular carcinoma through the miR-211-5p/CREB5 axis**

| **Name** | **sequence** |
| --- | --- |
| shNC | GATCCCCTTCTCCGAACGTGTCACGTTTCAAGAGAACGTGACACGTTCGGAGAATTTTT |
| shSNHG4-1 | GGCTCCACTAGGACACACAGATTTCAAGAGAATCTGTGTGTCCTAGTGGAGCTTTTT |
| shSNHG4-2 | GGAATGACATCTACCTCCATCATTCAAGAGATGATGGAGGTAGATGTCATTCTTTTT |
| SNHG4 | GCAGCAACGGAAAGGCGCCACGCTCGTGAGCGGAACCAGCGTTCCGGGGGCGCTCAGTGTGGGCAGGCAGGAAGCCTGGCTCCACTAGGACACACAGATTCTCTCCTGAGCAGCTGCGAACTATGCGCCCCTTCTACCCTTAAGAGATGGGATGGGAGTCCAACAAACCCAGCCATTGCTCAGACCCCAGCCCTTCTCTCCTCTAAGAAGCAGGTTCACCTCTGCCACCGCACTCGCATTTTTTTTTTTTTTTAAAGCCCGGCCTTTCCTAGGCGGGGTCAAGGGCCCCGCCCACCGAAGCCACGCCCAGTAGCCGCCCCGGGGCGGGGTTCCCCTCGGCTCCCGGCTGCCCTTTCCCCTCCGGCCTCTGCCGGTGCTGCTGCGCCCTGCGGAGCTCCGAACACGTGCGCAGAGGCTGGCTGTGGCAGATGCAACTGCAGGATGACTTGAAAGTAGGGCATCCTTCACCCATCTGAAGGGAGGAAATAGTGGCAGGTGACAGTCTGCATGTGCAGTTTTCAGATGCCTTCACCTGAATGACATCTACCTCCATCAGGACCCCAGATGTCTGACAGCCCTGTGTGACACCAAGATAAGTAACGTATGTAGTCTTCTTGTCATGTAGGTCCCAATTAAATTACTATAGCTCAGATGGGGGTAGGGGACTTAAAATTATGATGTGAAAAATTATGTAGAGTATCAGACTTTTTTTTGGGGGGGGACGGAGTCTTGCTCAGTTGCCCAGGCTAGAGTACAGTGGCTCGATCTCGGCTCACTGCAACCTCTGCCTCCTGGGTTCAAGCGATTCTCCTGCCTCAGCCTCCCGAGTAGCTGGGACTACAGGCACCTGCCACCATGCCAGGCTAATTTTTTTATTTTAAGTAGAGACGAGGTTTCACCATATTGGCCAGGCTGGTCTGAAACTCCTGACCTTGCGATTTGCCTGCCTTGGCCTCCCAAAATGCTGGGATTACAGGTTTTTTTGTTTGTTTTTTGAGACGGAGTCTCACTGTCTCCCAGGCTGGAGTGCAATGGCGCAATCTCGGCTCACTACAACCTCTACCTCCTGGGTTCAAGCGATTCTCCTGCCTCGGCCTCCT |
| CREB5 | ATGTTCTGCACCTCAGGAGGGAATTCAGCCTCAGTGATGTCCATGAGGCCTGTCCCAGGCTCTCTATCTTCTCTGCTACATCTCCACAACAGACAGAGACAGCCCATGCCAGCCTCCATGCCTGGGACCCTGCCCAACCCTACAATGCCAGGATCTTCCGCCGTCTTGATGCCAATGGAGCGACAAATGTCAGTGAACTCCAGCATCATGGGGATGCAAGGTCCAAATCTCAGCAACCCCTGTGCTTCTCCCCAGGTCCAGCCAATGCATTCAGAAGCCAAAATGAGGTTGAAGGCTGCATTGACTCACCACCCTGCTGCCATGTCAAATGGGAACATGAACACCATGGGACACATGATGGAGATGATGGGCTCCCGGCAGGACCAGACGCCACACCATCACATGCACTCGCACCCGCATCAGCACCAGACACTGCCACCCCATCACCCTTACCCACACCAGCACCAGCACCCAGCACACCATCCTCACCCTCAACCCCATCACCAGCAGAACCATCCACATCACCACTCCCATTCCCACCTTCATGCACACCCAGCACATCACCAGACCTCGCCACATCCGCCCCTGCACACCGGCAACCAAGCACAGGTTTCACCAGCAACACAACAGATGCAGCCAACCCAGACAATACAGCCACCCCAGCCCACAGGGGGGCGCCGGCGAAGGGTGGTAGACGAGGATCCGGACGAGAGGCGGCGGAAATTTCTGGAACGGAACCGGGCAGCTGCCACCCGCTGCAGACAGAAGAGGAAGGTCTGGGTGATGTCATTGGAAAAGAAAGCAGAAGAACTCACCCAGACAAACATGCAGCTTCAGAATGAAGTGTCTATGTTGAAAAATGAGGTGGCCCAGCTGAAACAGTTGTTGTTAACACATAAAGACTGCCCAATAACAGCCATGCAGAAAGAATCACAAGGATATCTAAGTCCAGAGAGTAGCCCTCCTGCTAGTCCTGTCCCAGCTTGCTCCCAGCAACAAGTCATCCAGCATAATACCATCACTACTTCCTCATCGGTCAGCGAGGTGGTAGGAAGCTCCACCCTCAGCCAGCTCACCACTCACAGAACAGACCTGAATCCGATTCTTTAA |
| si-NC | UAAGGCUAUGAAGAGAUAC |
| si-CREB5 | GCGGAAUAUCUCGAUGCAU |
| mimics NC | CCACUGUCCUGAAGUGAGAAA |
| miR-211-5p mimics | UUCCCUUUGUCAUCCUUCGC |
| inhibitor NC | CAGUACUUUUGUGUAGUACAA |
| miR-211-5p inhibitors | GCGAAGGAUGACAAAGGGAA |
